# Supplementary material for: The time‐of‐day of myocardial infarction onset affects healing through oscillations in cardiac neutrophil recruitment
Source: EMBO Mol Med. 2016 May 25;8(8):937–48. doi: 10.15252/emmm.201506083 (PMC4967945; doi:10.15252/emmm.201506083)
Supplement: Supplementary file 1 — Appendix [file EMMM-8-937-s001.pdf]

Appendix

The time of day of ischemia onset affects myocardial infarction healing and heart function through oscillations in neutrophil mobilization

Maximilian J. Schloss, cand. med.; Michael Horckmans, PhD; Katrin Nitz, MSc; Johan Duchene, PhD; ; Maik Drechsler, PhD[1, 3]; Kiril Bidzhekov, PhD[1]; Christoph Scheiermann, PhD; Christian Weber, MD; Oliver Soehnlein, MD, PhD; Sabine Steffens, PhD

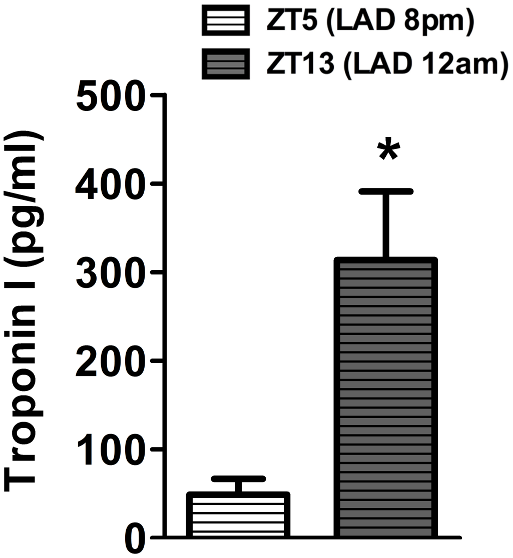

Appendix Figure S1: ZT13 MI leads to significantly increased cardiac damage compared to ZT5 MI in mice entrained to a shifted light cycle. Plasma troponin I levels 24 hours after MI. Student`s t-test; *N* = 5 for ZT5 MI and *n* = 6 for ZT13 MI; *P=0,0144.

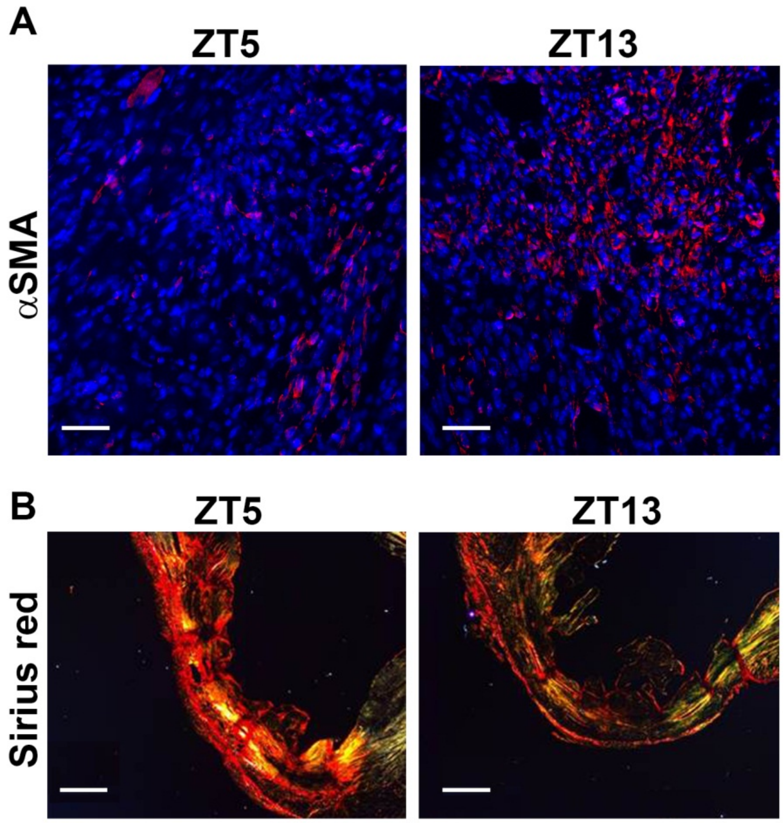

Appendix Figure S2: Histological analysis of myofibroblasts and collagen. MI was induced at ZT5 and ZT13 and hearts were harvested 7 days after ligation of LAD. **A,** Representative picture of myofibroblast staining within the infarct area using anti- alpha smooth muscle actin (SMA, positive red fluorescence) and 4′,6-Diamidin-2-phenylindol (DAPI) staining of nuclei (blue fluorescence); 20x magnification; Scale bar 50µm. **B,** Representative Image of Sirius-Red staining identifying collagen type I fibers as red fibers within the infarct area; 2.5x magnification; Scale bar 400µm.

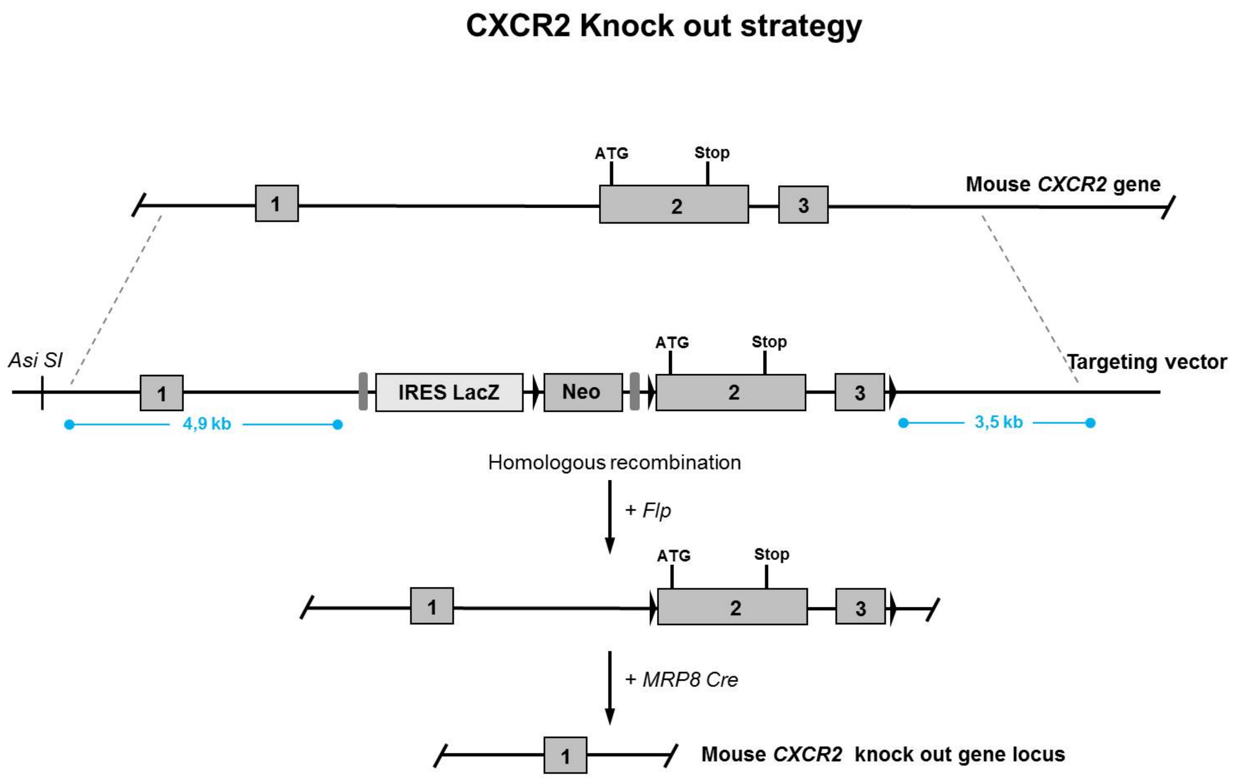

Appendix Figure S3: Generation of CXCR2-MRP8 Cre knockout mice. Schematic diagram of the generation of CXCR2 knockout mice. The exon-intron structure of the mouse CXCR2 locus is shown at the top. The targeting vector has a 4.9 kb 5′ arm including exon 1 and intron 1-2, the IRES-LacZ coding sequence, and a Neo selection cassette, both flanked by FRT sites (gray bars). The loxP sites (black triangles) flanking exons 2-3 (the coding part of CXCR2 gene) and the Neo gene. The 3′ recombination arm spanned 3.5 kb from the gDNA.

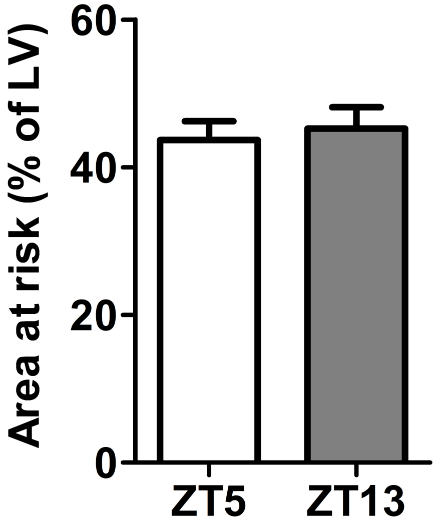

Appendix Figure S4: The area at risk is independent of the time of day of LAD ligation. Evan´s blue was injected into the left ventricle to distinguish between perfused cardiac tissue stained blue and non-perfused area at risk 24h after MI. The area at risk was calculated as the percentage relative to the left ventricle. *N* = 4 per group.

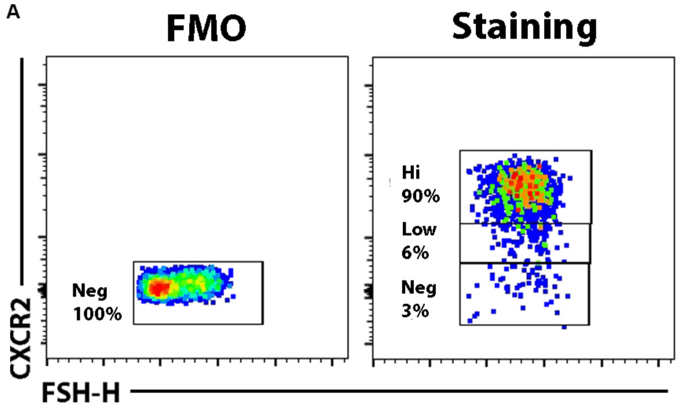

Appendix Figure S5: Gating strategy for CXCR2+ neutrophils in blood. The representative dot plots shows the gating strategy for CXCR2 expression on blood neutrophils (identified as CD45+Ly6G+CD11b+) at baseline (ZT5) gated as CXCR2^{high}, CXCR2^{low} and CXCR2^{neg} neutrophils.
